# Using U-Net convolutional neural network to model pixel-based electrostatic potential distributions in GaN power MIS-HEMTs

**DOI:** 10.1038/s41598-024-58112-9

**Published:** 2024-04-08

**Authors:** Bang-Ren Chen, Yu-Sheng Hsiao, Wei-Cheng Lin, Wen-Jay Lee, Nan-Yow Chen, Tian-Li Wu

**Affiliations:** 1 International College of Semiconductor Technology, National Yang Ming Chiao Tung University, Hsinchu, Taiwan; 2 Institue of Pioneer Semiconductor Innovation, National Yang Ming Chiao Tung University, Hsinchu, Taiwan; 3 National Center for High-Performance Computing, Hsinchu, Taiwan; 4 Institue of Electronics, National Yang Ming Chiao Tung University, Hsinchu, Taiwan

**Keywords:** GaN HEMT, Electrostatic potential modeling, Machine learning, U-Net, Engineering, Nanoscience and technology

## Abstract

This study demonstrates a novel use of the U-Net convolutional neural network (CNN) for modeling pixel-based electrostatic potential distributions in GaN metal–insulator-semiconductor high-electron mobility transistors (MIS-HEMTs) with various gate and source field plate designs and drain voltages. The pixel-based images of the potential distribution are successfully modeled from the developed U-Net CNN with an error of less than 1% error relative to a TCAD simulated reference of a 500-V electrostatic potential distribution in the AlGaN/GaN interface. Furthermore, the modeling time of potential distributions by U-Net takes about 80 ms. Therefore, the U-Net CNN is a promising approach to efficiently model the pixel-based distributions characteristics in GaN power devices.

## Introduction

Gallium nitride high electron mobility transistors (GaN HEMTs) are attractive due to two-dimensional electron gas (2DEG), large breakdown E-field, and the wide bandgap. Recently, thanks for the insulator under the gate metal, high performance power metal–insulator-semiconductor high electron mobility transistors (MIS-HEMTs) have been demonstrated^1–3^, which is promising for the power switching applications. However, the high electrical filed in the access region during an off-state drain bias stress can lead to the earlier breakdown, defect generations and trapping effects, limiting the performance and stability of these GaN HEMTs^4,5^. To address these issues, field plates (FPs) have been designed to optimize the electrostatic potential^6,7^; this is crucial for performance and reliability, particularly in GaN HEMTs for high-power applications. Numerical simulation techniques, such as technology computer-aided design (TCAD) simulations, have been widely used for modeling electrostatic potentials^8,9^. However, these simulations are time-consuming because of the complexity of the heterojunction and underlying physics. Moreover, although analytical models are often used to model device electrical characteristics^10,11^, analytical models cannot be efficiently applied to devices with different materials and structures.

Recently, high-performance computing has enabled the use of deep learning techniques to predict the electrical properties of devices from the input parameters^12–17^. However, since fully connected neural networks are not designed for image-related tasks, electrostatic potential modeling in previous studies has been limited to one-dimensional devices^12^. On the other hand, the convolutional neural network (CNN) based approach^18–27^ is also attractive for pixel-based image prediction and segmentation, which has been widely used in the biomedical sciences^21,22,24,25^ due to local connection and weight-sharing advantages. Still, the applications of CNN-based approach are limited in the semiconductor research. Recently, one study reported that the pixels of the two-dimensional landscape of low-voltage Si devices could be modeled by a CNN with a U-Net architecture^26^. Note that the advantage of using a U-Net CNN is that, in general, U-Net CNN requires only few images to have a relatively good results^23–27^. The network of U-Net is structured with a contracting path and an expansive path, forming its distinctive U-shaped architecture. The contracting path embodies a conventional convolutional network, utilizing a series of convolution applications, each paired with a rectified linear unit (ReLU) and a subsequent max pooling operation. This phase systematically diminishes spatial information while amplifying feature data. Conversely, the expansive pathway unites feature and spatial information by employing up-convolutions and merging them with high-resolution features obtained from the contracting path through concatenation. However, this work^26^ is limited to the dimensional variation with only considering the impact of on-state voltage that directly applied at the surface of the semiconductor with simplified symmetric device design that is not practical for the real device fabrication, which results from the relative compact and shallow network constructed by only 4 layers of contracting path and the expanding path. Furthermore, the impacts of the external dimensional variations, e.g., field plate variations on top of the semiconductor, on the potential distribution inside the semiconductor are not discussed^26^, which is more practical for the consideration of the semiconductor design.

In this work, we demonstrate U-Net CNN approach to model the pixel-based electrostatic potential image in GaN power MIS-HEMTs considering the different gate and source field plates and drain voltages, showing that the electrostatic potential can be accurately and effectively modeling by using the U-Net CNN approach. Our modified U-Net is consisted of 7 layers of the contracting path and 7 layers of the expanding path. Each contracting path is consisted of 2 convolutions with ReLU followed by a maxpooling function. Last two layers of the contracting path even followed by a dropout function in order to avoid the overfitting problem. On the other hand, each layer of the expanding path is consisted of a upsampling with ReLU, the concatenation with the data from the counterpart of the contracting path, and 2 convolutions with ReLU.

### Device schematic and typical electrical property

**Figure 1:**
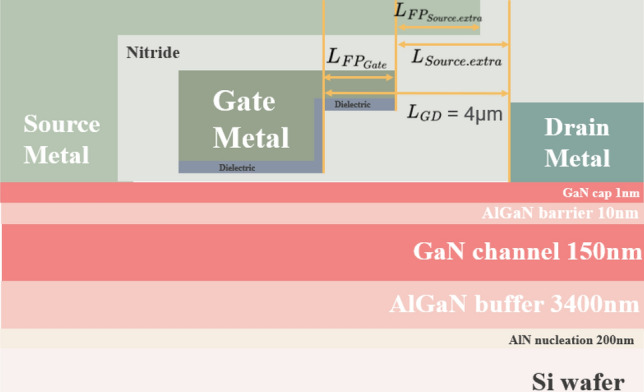
Schematic of the GaN MIS-HEMTs with the gate and drain field plate designs.

Schematic of a GaN MIS-HEMT is shown in Fig. 1. The electrostatic potentials in this work are generated by Sentaurus TCAD for various gate and source field plate designs and different applied drain biases ranged from 100 to 500 V with a step of 100 V. The gate and source field plates are designed by considering the ratio compared to the gate-to-drain distance (L_GD_) as shown in Eq. (1) & (2). Please note that the ratio of the gate and source field plates ranges from 0 to 1. The field plate designs used in this work are shown in Table 1.

1 $$Gate\;field\;plate\;ratio = \frac{{L_{{FP_{Gate} }} }}{{L_{GD} }}$$

2 $$Source\;field\;plate\;ratio = \frac{{L_{{FP_{Source.extra} }} }}{{L_{Source.extra} }}$$

**Table 1:** The field plate designs used in this work.

Gate field plate ratio	0.1	0.3	0.5	0.8
Source field plate ratio	0.1	0.3	0.5	0.8

**Figure 2:**
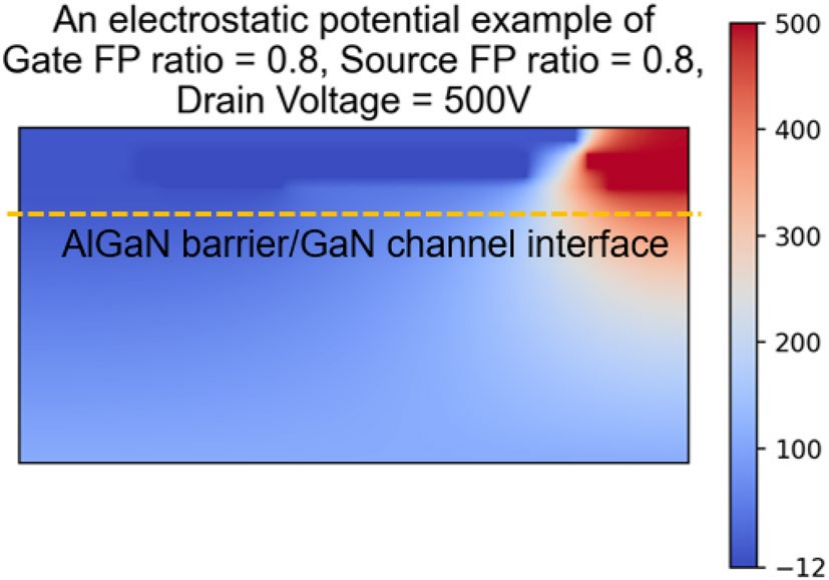
An example of the pixel-based images of electrostatic potential distribution generated from TCAD simulation.

A total of 80 images representing the electrostatic potential distribution were generated through TCAD simulations that ran for over 640 h. Figure 2 presents an example of the pixel-based images of electrostatic potential distribution generated from TCAD simulation with consideration of the gate FP ratio, source FP ratio, and drain voltage of 0.8, 0.8,and 500 V, respectively.

### Methodology

**Figure 3:**
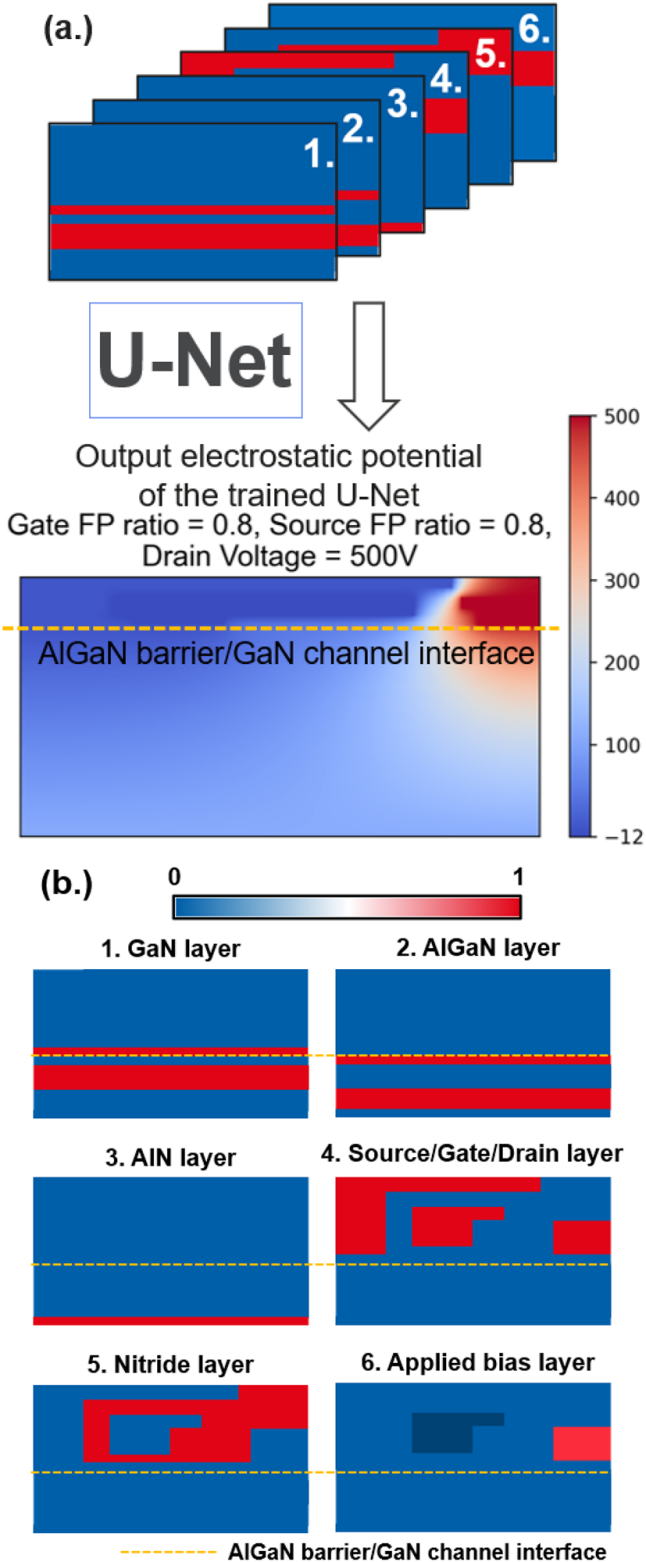
(**a**) An example of the electrostatic potential modeled by U-Net CNN and (**b**) the schematics of the 6 input layers.

Figure 3a presents the workflow for predicting the pixel-based electrostatic potential of the GaN MIS-HEMTs using a U-Net CNN model and schematics of the input layers. The images of device structures are divided into 6 layers to form the matrix representing the material information and applied voltage: (1) GaN, (2) AlGaN, (3) AlN, (4) source/gate/drain metal, (5) nitride, and (6) the applied bias (Fig. 3b). For layers 1–5 in the one-hot matrix, if the pixel location is the targeted materials, it will be recorded as “1” or “0” for not the targeted materials. Forthe 6^th^ layer, the voltage was linearly normalized to a range of 0–1.

**Figure 4:**
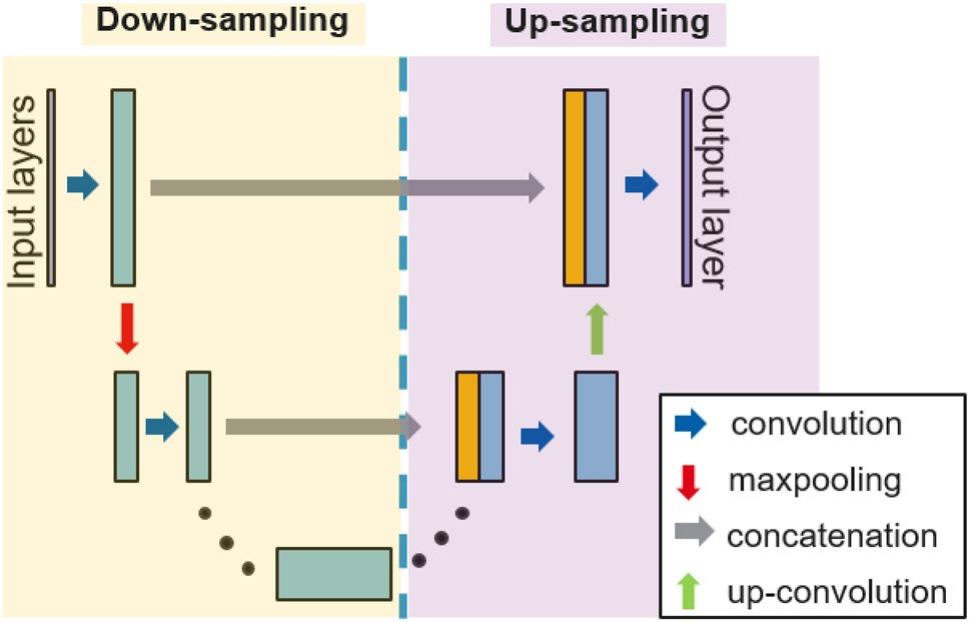
The simplified schematic of a U-Net model.

The U-Net CNN consists of two segments: the down-sampling side and the up-sampling side as shown in Fig. 4. The down-sampling side contains eight rows of convolution and maxpooling. The up-sampling side contains eight rows of up-sampling, convolution, and concatenation. The model is trained and evaluated by the mean-squared error (MSE) loss function, which measures the error between the output electrostatic potentials from the developed U-Net CNN and the reference electrostatic potential from the TCAD simulations. The goal of the training process is to minimize the loss function by adjusting its parameters, resulting in the better modeling. The optimizer for the iterations is Adam algorithm with an initial learning rate of 5 × 10^−6^. After the model is constructed, the images of the electrostatic potential distribution can be visualized for further verification.

The TCAD generated datasets are randomly divided into two groups: (1) the images for the training (72 images) and (2) the image for the verifications (8 images). The images for the verification purpose are not shown to the model during the training. Hence, they are suitable to verify the performance of the trained model.

## Results and discussions

**Figure 5:**
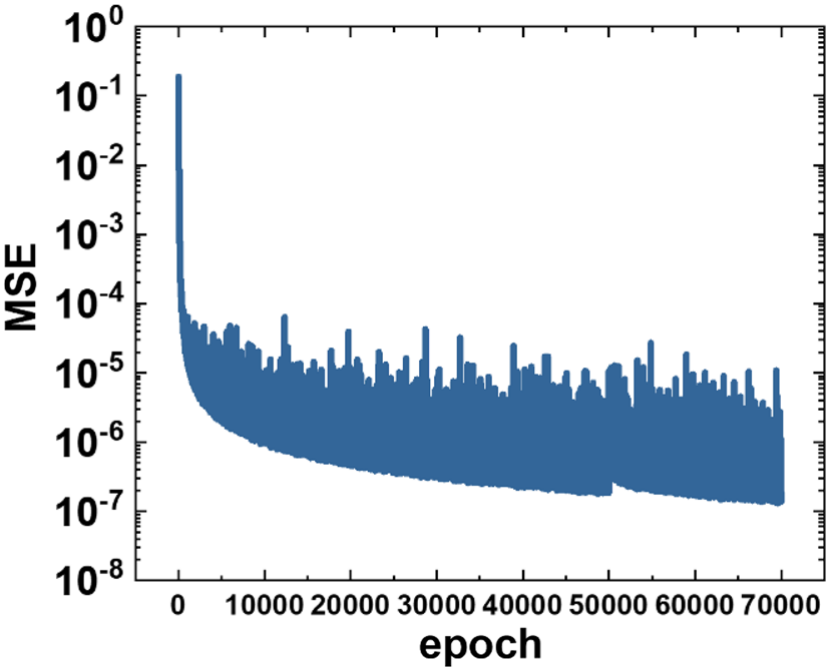
MSE records during the training process.

Figure 5 shows the decreases of the mean square error (MSE) during the training process, suggesting that developed U-Net CNN is efficient to minimize the error between the output electrostatic potentials from the developed U-Net model and the reference electrostatic potential from the TCAD simulations. Note that the TCAD computational environment is equipped with an 8 core Intel Xeon CPU, 47GiB of RAM, while the machine learning environment is equipped with a GPU of an Nvidia Tesla V100. Also, the training time of the U-Net CNN model is about 21.8 h.

**Figure 6:**
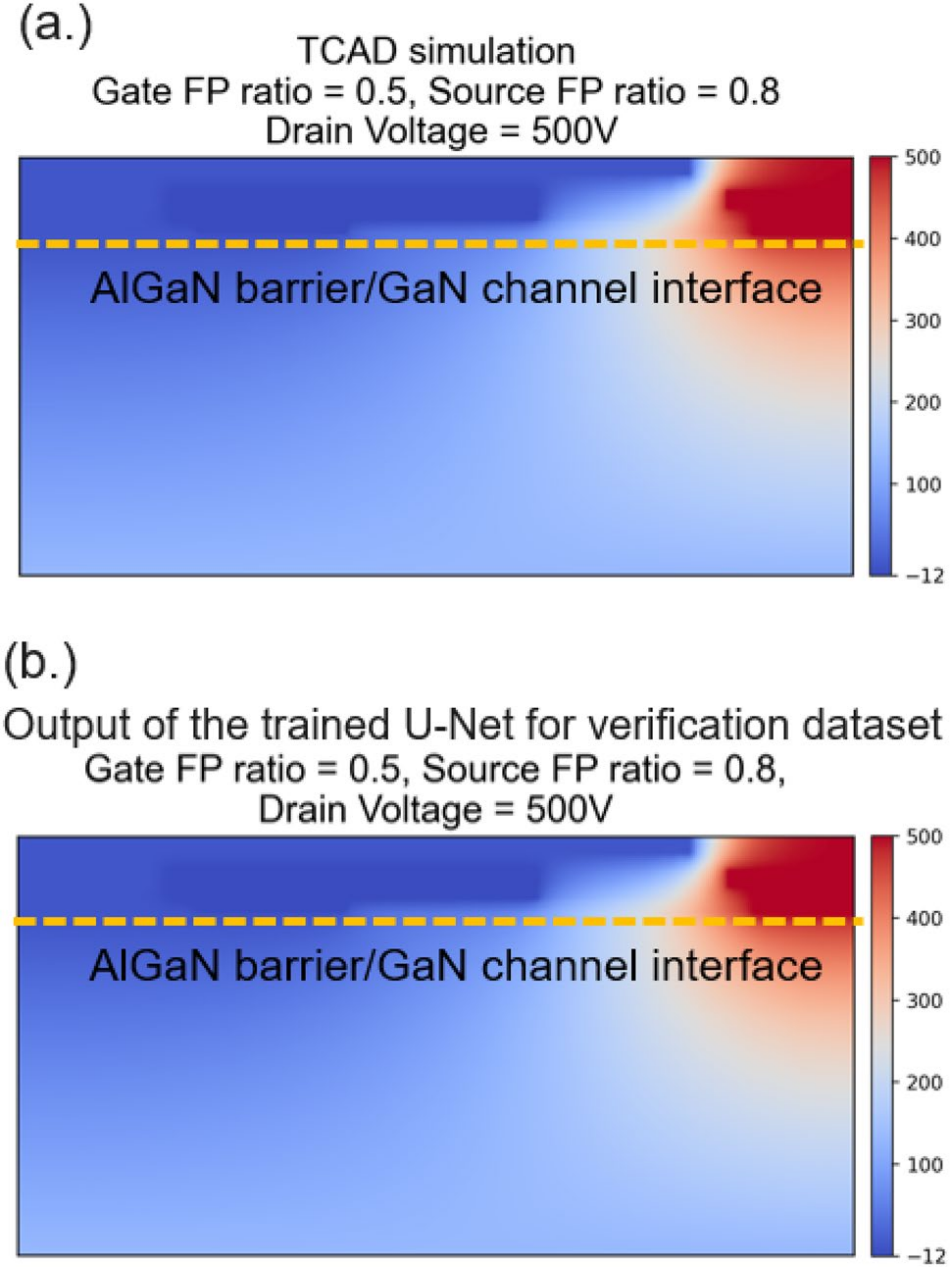
Comparison of electrostatic potential distributions from (**a**) the TCAD simulation and (**b**) the developed U-Net model.

**Figure 7:**
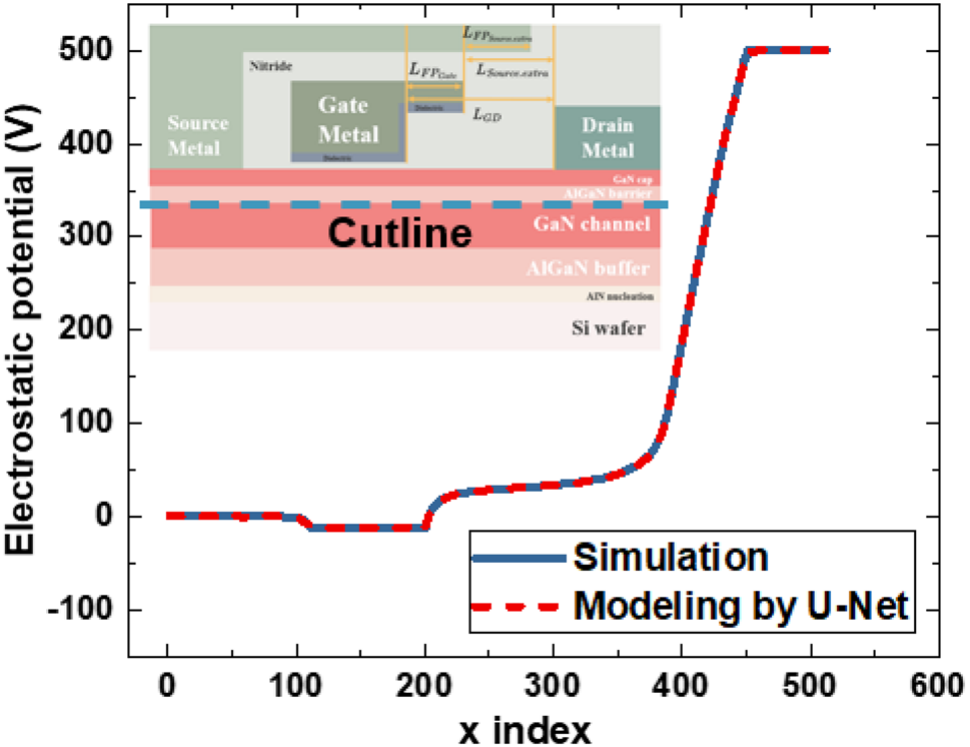
Comparison of electrostatic potential in AlGaN/GaN interface from the TCAD simulation and the developed U-Net model.

Figure 6 shows the side-by-side comparison of the simulation results from the TCAD and the modeling results (output) from the trained U-Net model in the dataset of the image for verifications. Furthermore, a less than 1% error of the electrostatic potential distribution in AlGaN/GaN interface can be obtained, indicating the excellent consistent results (Fig. 7). Furthermore, the modeling time of potential distributions by U-Net takes about 80 ms.

## Conclusion

In this work, the modeling of the electrostatic potential distributions is successfully demonstrated for the first time by considering the pixel-base images using the U-Net CNN with the different gate and source field plate designs and applied drain voltages in GaN power MIS-HEMTs. The different gate and source field plate designs and drain voltages are generated by TCAD simulation as references in this study, and the corresponding pixel-based electrostatic potential distribution images are modeled by the U-Net CNN. Less than 1% error of a 500 V electrostatic potential modeling in the AlGaN/GaN interface between the developed U-Net model and the reference electrostatic potential from the TCAD simulations can be achieved (error equation = (U_simulation_—U_U-Net_)/U_simulation_), indicating a successful modeling using developed U-Net CNN approach. Note that the modeling by the U-Net CNN requires only 80 ms, which is a huge speed improvement compared to the TCAD simulations. In sum, the U-Net CNN approach is promising for the accurate and efficient methodology in modeling the distribution-based characteristics, e.g., electrostatic potential, electric filed, current density, etc., in GaN power devices.

## Data Availability

Data inquiries can be directed to the corresponding author.
